# Assessment of Health-Related Quality of Life in Patients With Traumatic Fractures Following Thoracolumbar Fusion: Short-Term Results

**DOI:** 10.7759/cureus.53861

**Published:** 2024-02-08

**Authors:** Stylianos Kapetanakis, Nikolaos Gkantsinikoudis, Paschalis Tsioulas, Joanna Bladowska, Sotirios Apostolakis

**Affiliations:** 1 Department of Spine and Deformities, European Interbalkan Medical Center, Thessaloniki, GRC; 2 Department of Minimally Invasive and Endoscopic Spine Surgery, Athens Medical Center, Athens, GRC; 3 2nd Orthopaedic Department, General Hospital of Thessaloniki "Papageorgiou", Thessaloniki, GRC; 4 Faculty of Medicine, Wroclaw University of Science and Technology, Wroclaw, POL; 5 Department of Radiology, Wroclaw 4th Military Clinical Hospital, Wroclaw, POL

**Keywords:** quality of life, spinal fusion, vertebral fracture, lumbar spine, thoracic spine

## Abstract

Introduction: Thoracolumbar vertebral fractures (TVFs) constitute frequent injuries with specific therapeutic challenges and remarkable implications for affected individuals. The aim of this study is to investigate the alteration of overall health-related quality of life (HRQoL) in patients with traumatic TVFs undergoing thoracolumbar fusion surgery.

Materials and methods: A total of 72 patients with single-level traumatic thoracic or lumbar vertebral fractures (AO type A3 or A4) were enrolled in this prospective cohort study. All patients were subjected to thoracolumbar spinal fusion surgery with or without posterior decompression, being followed up for a two-year period. Clinical assessment was conducted via the implementation of the Visual Analog Scale (VAS) and 36-item Short-Form Survey Questionnaire (SF-36) for the evaluation of pain and HRQoL, respectively. Patient assessment was performed in determined postoperative follow-up intervals.

Results: Recorded values of assessed outcome measures demonstrated a statistically significant improvement during the entire two-year follow-up period. This improvement was more pronounced throughout the first three to six postoperative months, subsequently demonstrating a plateau. No statistically significant correlation between age, SF-36, and VAS was found, with the exception of the bodily pain index, the improvement of which was observed to be positively correlated with age. Transient causalgia and cerebrospinal fluid leak were recorded in 5% of evaluated individuals.

Conclusions: Thoracolumbar fusion constitutes a safe and efficient option for the surgical management of single-level traumatic vertebral fractures. Nevertheless, rehabilitation is a lasting procedure that may last over six months until final amelioration is observed. Clinical improvement may be more pronounced in older patients, potentially due to different expectations.

## Introduction

Traumatic vertebral fractures constitute a major cause of severe morbidity and mortality for young adults in Western societies, resulting in neurological compromise, intractable pain, deformity, and, most importantly, the undermining of quality of life. Fractures of the thoracolumbar spine, in particular, account for about 90% of all cases, representing usually the outcome of high-velocity motor vehicle accidents or falls from height [1,2].

The importance of systematic investigation of this condition and its complexity is illustrated by the numerous classification systems utilized to date, with the AOSpine system being currently the most widely accepted [3]. The decision of operative versus conservative treatment relies largely on these classification schemes with AO type B and C fractures requiring surgical stabilization and types A0, A1, and A2 fractures being treated invariably non-operatively [4]. Nevertheless, it is the A3 and A4 types that are also the most prevalent ones and for which clinical decision-making is more complex, and as such current literature has not reached a universal consensus. To our best knowledge, there are no available studies in existent literature, prospectively and multimodally assessing outcomes of surgical management of these fracture subtypes in patients with the absence of neurologic deficit and major systematic trauma, in the framework of quality of life analysis.

Hence, the aim of the present work is to investigate the surgical outcomes of spinal fusion in a cohort of patients with AO type A3 and A4 subtypes of vertebral fractures, particularly emphasizing on underlying health-related quality of life (HRQoL) in the context of a short-term follow-up analysis.

## Materials and methods

Patients and approvals

All patients enrolled in this study were subjected to open posterolateral fusion surgery due to traumatic fracture from January 2017 to December 2019. The primary aims and scope of this study were analytically explained to candidate participants, who decided to participate by signing a fully informed written consent. This study has obtained approval from the Institutional Review Board of Interbalkan European Medical Center, Thessaloniki, Greece (Approval Number: 28.12.2016). Furthermore, all distinct aspects of this study were in absolute concordance with ethical principles for medical research involving human subjects as defined in the Declaration of Helsinki of 1964 and its later amendments (2013).

Inclusion and exclusion criteria

Enrollment of patients in this study was decided according to specific predetermined inclusion and exclusion criteria. These criteria are analytically depicted in Table 1.

**Table 1:** Representation of study inclusion and exclusion criteria.

Inclusion criteria	Exclusion criteria
Single-level acute fracture of the thoracic or lumbar vertebral body classified as type A3 or A4 according to the established AOSpine thoracolumbar injury classification system [3]	Presence of multiple spinal fractures
Absence of neurological symptoms or associated deficit	
Severe central canal stenosis, according to imaginary evaluation, requiring posterior decompression	Presence of neurological symptomatology or neurologic deficit
Kyphotic deformity >15^o^	
Scoliotic deformity >10^o^	Age > 50 years
Age < 50 years	
No clinical or imaginary evidence of additional major trauma	Clinical or imaginary evidence of additional major trauma
Immobilization due to treatment-resistant pain	

Study design

All patients enrolled in this study were subjected to open posterolateral fusion with pedicle screw fixation from January 2017 to December 2019. All procedures were performed by the same experienced spine surgeon (KS) in two distinct tertiary centers. Operated patients were meticulously evaluated with standard predetermined outcome measures in regular chronic intervals preoperatively and at six weeks and three, six, 12, and 24 months postoperatively. Assessment was performed via clinical examination and implementation of established patient-reported outcome measures. Visual Analog Scale (VAS) and Short-Form 36 Medical Health Survey Questionnaire (SF-36) were recruited for assessment of perceived low back pain and underlying HRQoL, respectively. Evaluation of the data was performed independently by two experienced physicians. Any potential disagreement was resolved by mutual consensus and further evaluation by a third evaluator.

Surgical technique

**Figure 1:**
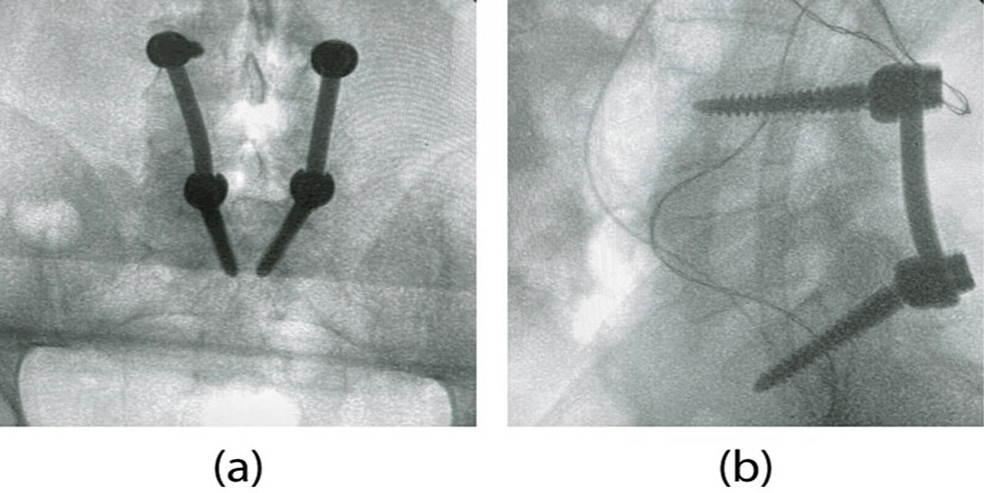
Anteroposterior (a) and lateral (b) intraoperative fluoroscopic images of a patient subjected to L3-L5 instrumentation for an L4 fracture.

All patients were subjected to preoperative evaluation with magnetic resonance imaging (MRI) and plain X-rays for surgical planning. A standard open posterolateral short-segment fusion with pedicle screw fixation was implemented in all individuals, as previously described [5]. In brief, following placement in the prone position, identification of the level of interest was performed via fluoroscopy in anteroposterior and lateral views. Paraspinal muscles were dissected subperiosteally and pedicle screws were placed under constant fluoroscopic guidance one level above and one below the fracture. Whenever indicated, posterior decompression through laminectomy and flavectomy was performed. Fusion was completed with the addition of 6 mm titanium rods and autologous bone graft in conjunction with osteoconductive putty (Figure 1). Immediately postoperatively, patients were closely monitored and mobilized on the first postoperative day with orthosis.

Postoperative care

Patients were instructed to wear a brace for a period of eight to 12 weeks, being subjected to intense physical therapy protocol with emphasis on strengthening core stabilizers (transversus abdominis, pelvic stabilizers, and quadratus lumborum) after the second postoperative week. In addition, they were generally instructed to refrain from lifting weights and performing extreme flexion, extension, and rotation of the torso.

Pain assessment

VAS constitutes a facile method for the evaluation of perceived pain intensity [6]. In this study, a unipolar horizontal line of 100 mm was used in all patients, who indicated the level of perceived pain with a marker in each sequential follow-up checkpoint. Recorded values were estimated in millimeters, implementing a one-decimal place approach. The level of minimal clinically significant alteration was defined at the level of 9 mm. No other related factors such as age, pain etiology, and gender were considered in the final evaluation [7].

Quality of life assessment

SF-36 represents a dominant patient-reported outcome measure for HRQoL in the field of spine surgery [8]. This questionnaire consists of 36 objects that multimodally evaluate eight parameters of an individual’s daily life: physical function (PF); role-physical (RP); bodily pain (BP); general health (GH); energy, fatigue, and vitality (V); social function (SF); role-emotional (RE); and mental health (MH). Higher percentages in these parameters are generally associated with favorable HRQoL.

Statistical analysis

Statistical analysis was conducted using STATISTICA 10.0 (StatSoft, Hamburg, Germany) and MATLAB 2016 (The MathWorks, Inc., Natick, MA). Figures were created using MATLAB 2016 (The MathWorks Inc.) and Adobe Illustrator CS3 (Adobe Systems, San Jose, CA).

The presence or not of normal distribution of recorded data was primarily assessed with the Shapiro-Wilk test. For non-parametric variables, chi-square, Mann-Whitney’s test, and Kruskal-Wallis H test were used to test for differences between two and multiple groups, respectively. When paired data were compared, the Wilcoxon matched pairs test and Friedman’s ANOVA were conducted. Spearman’s test was applied to examine for potential correlations between the parameters. In all cases, the level of statistical significance was p < 0.05. The Bonferroni correction for multiple comparisons was applied accordingly.

## Results

**Figure 2:**
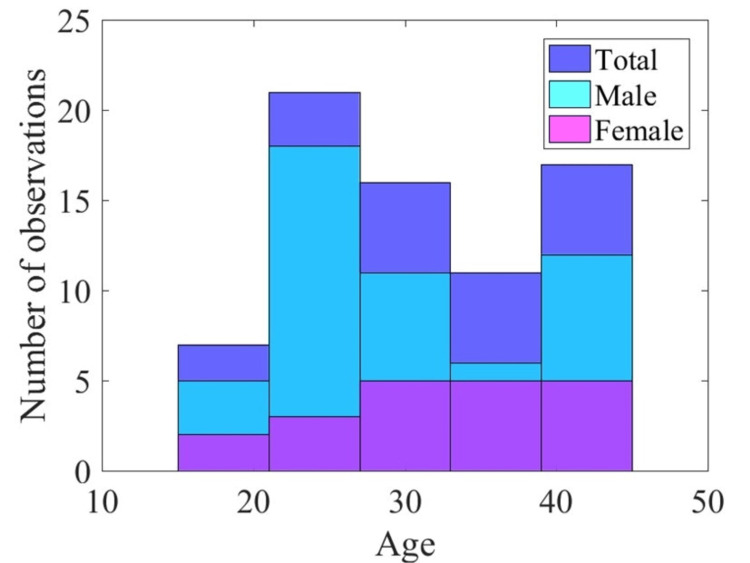
Schematic representation of age profiles of included patients.

In total, 72 patients were finally recruited in the study. Demographic characteristics of enrolled participants are presented in Table 2. The majority of the patients were in the third decade of life (Table 2 and Figure 2). A statistically significant difference in gender distribution from that of a theoretical random sample (X21, 72 = 7.48; p < 0.01) was found. The age profiles of the two groups were not found to differ significantly (U1, 75 = 431; p = 0.266).

**Table 2:** Demographic features of enrolled participants.

Feature	Value
Sample size (n)	72
Age (mean ± SD)	30.6 ± 8.4
Sex	
Male	52 (72.2%)
Female	20 (27.8%)
Fracture level	
T1-T4	4 (5.5%)
T5-T9	11 (15.3%)
T10-L2	39 (54.2%)
L2-L5	18 (25.0%)

All patients were subjected to uneventful open posterolateral short-segment fusion with pedicle screw fixation as already described, featuring no major perioperative complications. Three patients (4.1%) reported transient causalgia immediately postoperatively that resolved spontaneously prior to the first scheduled follow-up. One case (1.4%) of intraoperative CSF leak was identified and corrected on the first instance with no associated sequelae postoperatively. No other major or minor complications were observed. All enrolled individuals successfully reached the end of follow-up at two years postoperatively.

As far as the individual indices of SF-36 are concerned, a statistically significant difference in all indices was observed among the various evaluation stages. The same also applied to the VAS score for back pain with a median decrease of 80.5% (Table 3).

**Table 3:** Representation of values alteration of recorded indices during extreme follow-up checkpoints. PF: physical functioning; RP: role-physical; BP: bodily pain; GH: general health; V: vitality; SF: social function; RE: role-emotional; MH: mental health; VAS: Visual Analog Scale.

Index	Median preoperative value	Median 2-year postoperative value	ANOVA
PF	34	82.5	F = 0.98, P under 0.001
RP	36	82	F = 0.99, P under 0.001
BP	35	82	F = 0.99, P under 0.001
GH	33.5	82	F = 0.98, P under 0.001
V	34	83	F = 0.99, P under 0.001
SF	35	82	F = 0.98, P under 0.001
RE	34	83	F = 0.99, P under 0.001
MH	34.5	82	F = 0.98, P under 0.001
VAS	88	18	F = 0.95, P under 0.001

**Figure 3:**
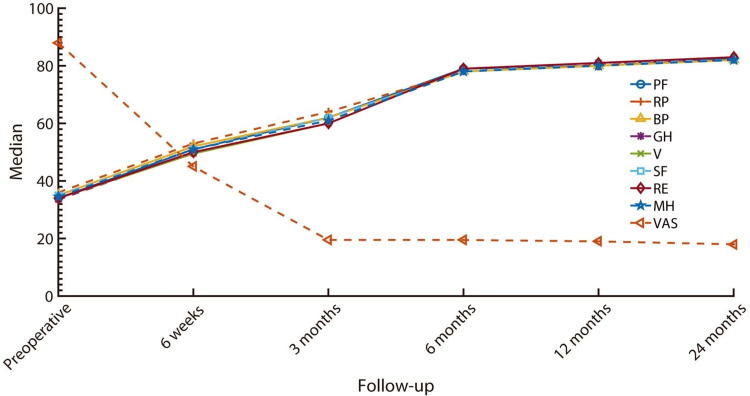
Median values of VAS and individual indices of SF-36 over the two-year follow-up period. PF: physical functioning; RP: role-physical; BP: bodily pain; GH: general health; V: vitality; SF: social function; RE: role-emotional; MH: mental health; VAS: Visual Analog Scale; SF-36: 36-Item Short-Form Survey Questionnaire.

Post-hoc analysis of the various time periods revealed a continuous improvement of all indices of SF-36 and VAS over the entire follow-up period (p < 0.001; Figure 3).

**Figure 4:**
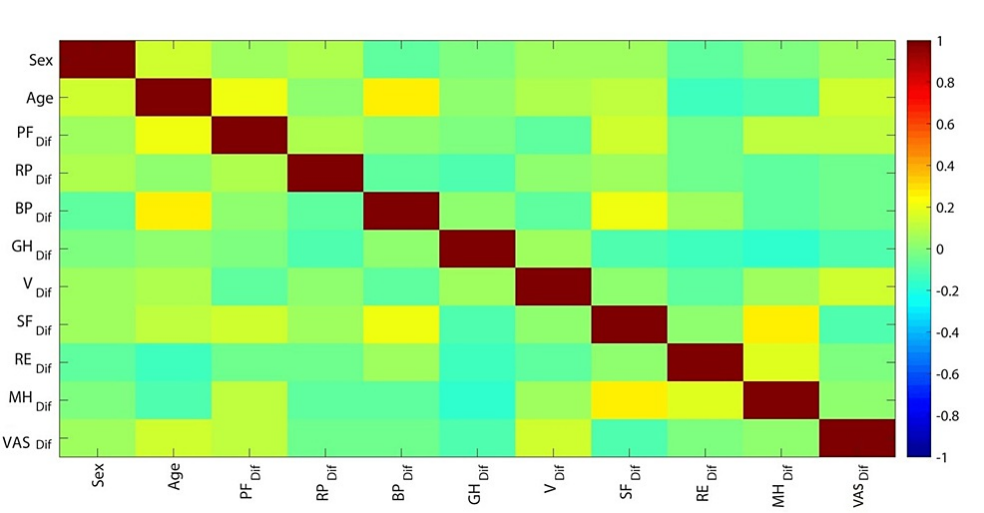
Correlation matrix of the study's main outcome measurements. PF: physical functioning; RP: role-physical; BP: bodily pain; GH: general health; V: vitality; SF: social function; RE: role-emotional; MH: mental health; VAS: Visual Analog Scale.

In addition, the improvement in all indices was found to be irrespective of age and gender, with the exception of the bodily pain index of SF-36, the improvement of which was found to be positively correlated with age (r = 0.27, p < 0.01) (Figure 4).

## Discussion

Herein, we report the assessment of the HRQoL in patients who underwent open posterolateral fusion with pedicle screw fixation for single-level traumatic vertebral fracture. Our initial hypothesis of the ameliorative role of fusion surgery in individuals with AO type A3/A4 fractures was confirmed post-analysis of evaluated outcome measures. The different clinical manifestations of vertebral fractures inevitably impose confounding factors when assessing functional status. Hence, in comparison to previous studies addressing the same topic, postoperative outcomes are investigated in a strictly defined subset of trauma patients and in a systematic way over a two-year period. On top of this, several orthopedic fractures frequently coexist, rendering distinction among the causes of discomfort arduous in the vast majority of affected individuals [9].

In concordance with the results of previous investigations [10,11], our study demonstrated that traumatic thoracolumbar spine fractures represent high-energy injuries that are observed more frequently in male individuals. Among the over 3100 patients included in the study by Wang et al. [10], 65.5% were men, while another study found a male-to-female ratio of 5.87 [12]. In all of the aforementioned studies, the greatest incidence was found in adults in the fourth decade of life. This observation underlines the great socioeconomic burden that this condition imposes, alongside the necessity for the development of an effective treatment that will not only decrease morbidity but also allow rapid rehabilitation and timely return to work. In fact, trauma patients with unstable fractures spend over 20 days in hospital, a hospitalization associated with remarkable socioeconomic sequelae [13]. Considering the cases of vertebral fractures with concomitant spinal cord injury, the mean absence from work is over 100 days, with 13% of patients being unemployed within one year from injury [14]. Another relative study reported that only about half of the patients returned to their previous level of employment [15].

Investigating the etiology of these fractures, a higher prevalence of female sex in low-impact injuries, such as falls from under 2 m and sports injuries, has been found. Across the entire spine, the most frequent location of fracture is the thoracolumbar junction, potentially due to its higher mobility [10].

As far as the primary goals of this study are concerned, a median increase in all indices of SF-36 of at least 133% was observed, with a concurrent decrease in VAS score by 80.5% within the two years of follow-up. Observed amelioration is considerably quantitatively greater than those reported in previous studies, possibly due to patient selection [5]. While an improvement in all functionality parameters was observed between consecutive time periods, improvement rates were depicted to be maximal at six-month intervals, demonstrating a subsequent plateau until the end of follow-up.

Previous studies have indicated that following posterior instrumentation, patients return to the level of disability and enjoy a quality of life similar to that of uninjured individuals [16]. Nevertheless, other groups of scholars challenge this claim [17]. This disagreement is potentially patient-related, as several predictors of outcome in the management of low-back pain have been established, including sex, age, and lifestyle factors [18-20]. Regarding the operative modality used, no statistically significant difference has been found in the SF-36 scores between the minimally invasive and the conventionally open groups [21]. The same were the conclusions of another research group when comparing SF-36 and VAS scores between operative and non-operative treatments [22].

Further extrapolating our investigation, we aimed to examine the potential correlation of parameters of interest with particular demographic features of enrolled patients. The positive correlation between the BP index of SF-36 and age could reflect the higher expectations of older patients from their operation. Reduced need for mobilization, life habits, and pain tolerance may collectively have played a major role in the greater improvement in senior patients. To the best of the authors’ knowledge, such a correlation has not been investigated in previous studies, and as such, further investigation is warranted.

Besides the assessment of HRQoL through a structured questionnaire, postoperative outcomes have also been reviewed through radiological evaluation. Post-traumatic kyphotic deformity is a major cause of morbidity, due to loss of sagittal balance and concurrent aesthetic concerns. Open fixation has not been found to be superior to the respective percutaneous when examining Cobb angle correction [23]. A recent meta-analysis also failed to demonstrate any significant difference in the Cobb angle between anterior and posterior approaches [24]. Some authors support the removal of the prosthesis about one year following the accident, as sufficient alignment has been already achieved [25]. Despite being of great importance, such an investigation was beyond the scope of the present article.

Finally, a complication rate of 11.7% for posterior short-segment fusion has been documented in a systematic review by Verlaan et al. [26]. Implant malposition, early implant failure, aseptic bone necrosis, and dural tears were the most frequently observed complications. Furthermore, 23.9% of patients needed to be transfused within 90 days of surgery [27]. Importantly, in our study, no such cases were documented. Nevertheless, these complications are demonstrated to be more frequent in particular patient subgroups such as patients with ankylosing spondylitis, with a large proportion of them experiencing major adverse events and a 90-day mortality of as high as 36% [28].

Despite that, in our study, in conformity with relative investigations in the available literature, no patients with neurologic deficits were enrolled, thoracolumbar vertebral fractures (TVFs) may be associated with remarkable neurologic sequelae as a result of spinal canal compromise with the spinal cord, dural sac, and nerve root impingement. Regarding lower lumbar levels, TVFs with posterior wall involvement and displacement of bone fragments may be related to cauda equina syndrome (CES), an emergent condition necessitating direct surgical management to avoid permanent neurologic decline and quality of life undermining. CES is typically recognized with severe lower back pain, bilateral acute lower limb weakness in conjunction with saddle anesthesia, and urinary/fecal incontinence. Surgical management of CES in the acute trauma setting may be challenging, especially in cases with dural tear, nerve root injury, and excessive kyphotic deformity of affected vertebra [29]. In these conditions, as also occurs with CES as a result of disc herniation, prompt surgical management is required [29,30]. Furthermore, except for timing, other factors such as age and affected level may significantly influence functional outcomes and should be seriously considered [30].

Despite the aforementioned data, the results of our study should be interpreted with caution since specific underlying limitations may hinder their generalization. First, this study reports results from a prospective investigation in two distinct tertiary centers, not including a control group in the framework of a potential multicenter randomized controlled trial. Second, the number of enrolled individuals as well as follow-up duration may be considered limited, in the effort to clearly delineate the pure effects of thoracolumbar fusion surgery in patients with single-level traumatic vertebral fractures of the thoracolumbar spine. Third, it should be also stated that this study veritably reflects a single-surgeon series, with significant experience in spinal fusion surgery. Last but not least, it is also crucial to highlight that patients with multiple spinal fractures or signs of additional major organ trauma, both representing frequent conditions in trauma, were excluded from this investigation. Nevertheless, the aim of this investigation was to provide our short-term multifaceted results of fusion surgery in adult patients with single-level traumatic thoracolumbar fractures, particularly focusing on perceived pain and quality of life status. Undoubtedly, the results of our study warrant further investigation in the framework of large well-designed multicenter randomized controlled trials.

## Conclusions

In conclusion, our results demonstrated that open posterolateral fusion surgery with pedicle screw fixation with or without spinal canal decompression represents a safe and effective option for clinical management of traumatic thoracolumbar fractures. However, patients should be aware in advance that the rehabilitation period is long and can last as long as six months. Following that, a significant yet less profound improvement in quality of life can be observed. Regardless of the significance of these results, further studies are required to provide a deeper insight into the factors that determine the quality of life of patients following traumatic vertebral fractures.
